# Survival and division fate programs are preserved but retuned during the naïve to memory CD8^+^ T‐cell transition

**DOI:** 10.1111/imcb.12699

**Published:** 2023-10-15

**Authors:** Susanne Heinzel, HoChan Cheon, Gabrielle T Belz, Philip D Hodgkin

**Affiliations:** ^1^ Immunology Division Walter and Eliza Hall Institute of Medical Research Parkville VIC Australia; ^2^ Department of Medical Biology The University of Melbourne Parkville VIC Australia; ^3^ Frazer Institute The University of Queensland Brisbane QLD Australia

**Keywords:** CD8^+^ memory T cells, cytokine sensitivity, T cell proliferation dynamics, T cell response dynamics

## Abstract

Memory T cells are generated from naïve precursors undergoing proliferation during the initial immune response. Both naïve and memory T cells are maintained in a resting, quiescent state and respond to activation with a controlled proliferative burst and differentiation into effector cells. This similarity in the maintenance and response dynamics points to the preservation of key cellular fate programs; however, whether memory T cells have acquired intrinsic changes in these programs that may contribute to the enhanced immune protection in a recall response is not fully understood. Here we used a quantitative model–based analysis of proliferation and survival kinetics of *in vitro*–stimulated murine naïve and memory CD8^+^ T cells in response to homeostatic and activating signals to establish intrinsic similarities or differences within these cell types. We show that resting memory T cells display heightened sensitivity to homeostatic cytokines, responding to interleukin (IL)‐2 in addition to IL‐7 and IL‐15. The proliferative response to αCD3 was equal in size and kinetics, demonstrating that memory T cells undergo the same controlled division burst and automated return to quiescence as naïve T cells. However, perhaps surprisingly, we observed reduced expansion of αCD3‐stimulated memory T cells in response to activating signals αCD28 and IL‐2 compared with naïve T cells. Overall, we demonstrate that although sensitivities to cytokine and costimulatory signals have shifted, fate programs regulating the scale of the division burst are conserved in memory T cells.

## Graphical abstract

In this study, we found that murine memory T cells, upon activation, initiate a timed division burst followed by an autonomous return to quiescence that is identical in timing and scale to that described for naïve T cells. Despite these similarities in cellular fate programming, memory T cells have acquired different sensitivities to cytokine and costimulatory signals. They show a heightened sensitivity to homeostatic cytokines, while upon antigen stimulation they are less sensitive to cytokine and costimulatory signals.

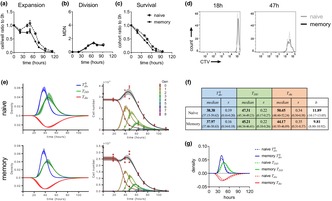

## INTRODUCTION

Immunological memory forms an important part of the adaptive immune system, providing faster and more efficient protection when challenged with a previously encountered pathogen.^1^ Memory T cells are formed during the initial immune response. In the initiating event, naïve T cells respond with a rapid division burst and generate a large pool of antigen‐specific effector cells and a smaller pool of memory cells. Upon clearance of infection, the population of effector cells contracts by apoptosis, leaving behind a small pool of long‐lived, antigen‐specific memory cells. The ability to maintain these memory cells for an extended period (years)^2^ and for the cells to undergo rapid activation, expansion and development into effector cells following re‐exposure to the same pathogen is a key feature underlying the efficacy of immunological memory. Furthermore, upon reactivation, new memory cells are generated, enabling persistent maintenance of immunity for long periods.^3^, ^4^, ^5^, ^6^, ^7^

How the recall response to secondary infection is accelerated over the primary response has been a matter of debate. Given their central role in immune protection, the response kinetics of memory T cells have been extensively studied *in vitro* and *in vivo*, often with conflicting results. One accepted difference is that memory T cells are poised and acquire effector functions more rapidly than naïve cells upon stimulation.^8^ This difference is due to epigenetic remodeling of the chromatin structure in effector gene loci during the primary activation event.^9^ However, there have been conflicting reports on whether the accelerated secondary immune response is due to intrinsic changes in activation thresholds or proliferation kinetics. Several reports have demonstrated a faster and greater expansion of memory T cells *in vitro* and *in vivo*,^8^, ^10^, ^11^, ^12^, ^13^, ^14^ while others have shown a more rapid response in naïve T cells.^15^, ^16^ Whether or not a naïve T cell is recruited into the immune response is controlled by the level and affinity of the T‐cell receptor (TCR) signal and the presence and level of cytokines and costimulatory signals.^17^, ^18^, ^19^ Both increased^20^ and decreased^21^, ^22^ antigen thresholds required for activation have been reported for memory T cells. A further point of difference noted in some studies is sensitivity to costimulation. Early studies have indicated that memory cells might be costimulation independent. However, more recent studies provide evidence that memory T cells benefit from costimulation in a manner analogous to naïve cells.^23^, ^24^, ^25^, ^26^

Despite these differences, the shared features of long‐term maintenance of quiescent cells and the proliferative burst and generation of effector and memory T cells in response to antigenic stimulation point toward conserved cellular fate programs in naïve and memory T cells.

Here we return to the question of intrinsic differences and similarities between naïve and memory cells by applying new quantitative methods to isolate and measure kinetic differences in critical cellular fate timers. The framework we apply assumes that similar cells exhibit stochastic differences in either their construction or their history that alter their response to their environment or stimulation and can be described with appropriate probability distributions. Further, it assumes that cells respond to stimulation to modify internal fate timers and proliferate and survive according to simple rules that can be measured with quantitative comparisons or the fitting of suitable mathematical models. These underlying assumptions are supported by tracking the division history of clonal families by flow cytometry and long‐term video imaging. Such studies reveal that stimulated T‐cell clones, as seen for B cells, divide symmetrically for a period and then, by default stop dividing, return to quiescence and die.^27^, ^28^, ^29^ Notably, the programmed return to quiescence at the termination of the division burst, termed “division destiny,” has the characteristics of a timed process and is governed by expression and loss of oncoprotein Myc.^30^ The time division destiny is reached is a function of the signals received *via* the TCR, costimulation and cytokines and can be extended by ongoing signals from cytokines.^28^, ^30^, ^31^

We used carefully controlled *in vitro* stimulation conditions to measure survival and proliferation dynamics in response to cytokines, antigen and costimulatory signals to establish cellular fate programming and compare naïve and memory cell sensitivity. We used a combination of precursor cohort methods that directly compare dynamics^30^, ^32^, ^33^ and, where helpful, fitted time‐series data with the probabilistic Cyton2 model based on the independent regulation of times to divide, reach destiny and die.^27^ We found that fundamental fate programs controlling survival and division in response to antigenic stimulation are maintained in memory CD8^+^ T cells. While our comparison of response rates between naïve and memory T cells revealed strong similarities, there were clear cellular programming differences affecting sensitivities to cytokines and costimulation.

## RESULTS

### Resting memory and naïve T cells vary in sensitivity to IL‐7, IL‐15 and IL‐2

As lymphocytes rely on signals provided by cytokines for their homeostatic survival, the range of signals detected and their relative sensitivities are a potential point of difference between naïve and memory cells.^34^ To explore such differences in preparation for studying changes after activation, we measured the intrinsic life span of resting naïve and memory CD8^+^ T cells cultured *ex vivo* with and without survival‐promoting factors, interleukin (IL)‐7, IL‐2 and IL‐15. To generate memory cells for these experiments, naïve OT‐I CD8^+^ T cells were transferred to C57BL/6 mice 1 day before infection with *Listeria‐OVA*. Several weeks after infection was cleared, memory CD8^+^ OT‐I T cells were isolated and compared with CD8^+^ T cells from nonimmunized OT‐I mice.

**Figure 1:**
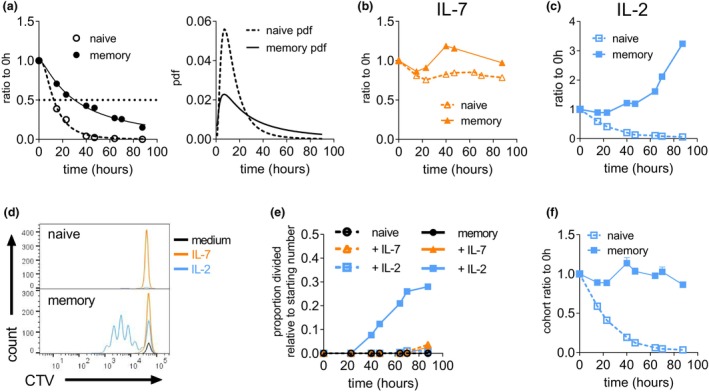
Population dynamics of resting naïve and memory CD8^+^ T cells in response to interleukin (IL)‐7 or IL‐2. Memory OT‐I CD8^+^ cells (filled symbols) were isolated 10 weeks after transfer into C57BL/6 mice and infection with *Listeria‐OVA* (total age of OT‐I cells was about 16 weeks) and compared with naïve OT‐I CD8^+^ cells (open symbols) isolated from 17‐week‐old nonimmunized mice. Cells were cultured in complete media alone, or the presence of 1 ng mL^−1^ IL‐7 or 100 U mL^−1^ recombinant human IL‐2. **(a)** Number of naïve and memory T cells relative to starting cell number cultured in medium alone, fitted to log‐normal survival curve as in the right panel, **(b)** with IL‐7 or **(c)** with IL‐2. **(d)** Representative CellTrace Violet (CTV) division plots measured at 87 h after the start of culture. **(e)** The proportion of cells of the starting population that have undergone at least one division over time. **(f)** The proportion of cohort number relative to starting population of cells cultured with IL‐2. Data are presented as means ± standard error of the mean of triplicate cultures. Data are representative of three independent experiments.

Tracking death rates of unstimulated, resting naïve and memory OT‐I CD8^+^ T cells showed that the latter population possessed greater intrinsic survival potential. The rate of death conformed to log‐normal survival curves and indicated the time for 50% of cells to be lost, taking 21 h longer than naïve cells (Figure 1a). As expected, IL‐7 promoted strong survival in both naïve and memory CD8^+^ OT‐I cells, with cell numbers remaining stable for over 90 h of culture (Figure 1b). However, the response to IL‐2 differed sharply between the two groups. The addition of IL‐2 had little effect on resting, unstimulated naïve T cells (Figure 1c). By contrast, the number of resting memory CD8^+^ T cells not only remained constant during the early phases of the culture but also increased as a result of the strong induction of cell division (Figure 1c, d), with about 30% of the starting cell population having undergone at least one division at 90 h of culture (Figure 1e). Transforming the cell numbers by precursor cohort analysis removed the effect of proliferation and isolated the survival effects.^30^, ^32^, ^35^ This plot revealed that in addition to promoting mitosis, IL‐2 provided a strong survival stimulus for resting memory T cells, with no cell loss observed across divided and nondivided cells for the duration of the experiment (Figure 1f).

Furthermore, as expected,^34^ memory CD8^+^ T cells were much more sensitive to survival signals conferred by IL‐15 (Supplementary figure 1a–c). However, in contrast to IL‐2, no cell division was induced by IL‐15 in resting memory cells (Supplementary figure 1d) despite these cytokines sharing the beta and gamma chains of their respective receptors. Differences in IL‐2 and IL‐15 sensing and responsiveness by T cells have been previously reported and traced to protein synthesis and cell growth.^36^

These results demonstrate distinct response patterns of resting naïve and memory CD8^+^ T cells: while naïve cells are limited to IL‐7, memory T cells can respond to several cytokines to maintain their cell numbers and, as was observed in the presence of IL‐2, are even able to expand in the absence of specific antigenic stimuli.

### Loss of sensitivity to homeostatic cytokines after TCR stimulation is conserved in memory T cells

We next examined kinetic events following TCR‐mediated activation, starting with the reprogramming of cell survival. We had shown previously that upon stimulation, naïve T cells actively transition their dependence for survival from cytokines, such as IL‐7‐promoting Bcl‐2, to TCR‐driven survival mediated by A1 and Bcl‐xL.^37^ Furthermore, the TCR‐mediated signal directly suppresses sensitivity to IL‐7, thus removing the reliance on Bcl‐2.^37^

As sensitivity to IL‐7 is regained during the differentiation to memory T cells (refer Surh and Sprent^34^ and Figure 1), we examined whether survival regulation in response to TCR signaling was equivalent to naïve T cells. To investigate this, we first used CD44^high^ memory CD8^+^ T cells from C57BL/6 mice isolated 11 weeks after infection with *Listeria‐OVA*. The effect of autocrine‐produced IL‐2 was eliminated by the addition of the mouse IL‐2 neutralizing antibody clone S4B6, which *in vitro* blocks the action of mouse IL‐2.^19^

**Figure 2:**
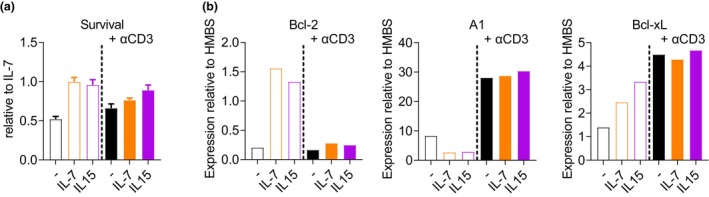
T‐cell receptor (TCR) signaling induces a switch of intrinsic survival program in memory CD8^+^ T cells. CD44^+^CD8^+^ memory T cells were isolated from C57BL/6 mice 11 weeks after infection with *Listeria‐OVA*, CellTrace Violet labeled and cultured for 24 h in complete media (open bars) or stimulated with plate‐bound αCD3 (filled bars) without cytokines, or in the presence of interleukin (IL)‐7 or IL‐15. **(a)** Total cell number relative to cells cultured in IL‐7 as an estimate for maximum survival: IL‐7, 2 ng mL^−1^ and IL‐15, 2 ng mL^−1^. **(b)** RT‐qPCR analysis of Bcl‐2, Mcl‐1, A1 and Bcl‐xL messenger RNA. IL‐7, 10 ng mL^−1^ or IL‐15, 10 ng mL^−1^. Data in **a** are presented as means ± standard error of the mean of triplicate cultures. Data in **a** are representative of two (conditions with IL‐15) or three (all other conditions) independent experiments. Data in **b** are representative of two (conditions with IL‐15) or three (all other conditions) independent experiments.

Similar to observations in naïve T cells,^37^ *in vitro* stimulation with αCD3 led to a reduction of memory T cells within the first 24 h (Figure 2a). This loss was not rescued by the addition of IL‐7, indicating a loss of sensitivity to this cytokine through TCR engagement in memory T cells, whereas IL‐15 provided a slightly greater survival advantage than IL‐7 for αCD3‐stimulated memory T cells.

Bcl‐2, A1 and Bcl‐xL messenger RNA were measured to investigate whether TCR stimulation induced the same reprogramming as has been described for naïve T cells.^37^ As expected, both IL‐7 and IL‐15 upregulated Bcl‐2 messenger RNA in resting memory cells (Figure 2b). However, the production of Bcl‐2 was almost completely abolished by αCD3 stimulation even in the presence of IL‐7 and IL‐15. At the same time, induction of an alternative survival program was evident by strong induction of A1 and Bcl‐xL (Figure 2b). The loss of Bcl‐2 production immediately after TCR signaling was confirmed in antigen‐specific OT‐I CD8^+^ memory T cells isolated 3 months after infection with *Listeria‐OVA* (Supplementary figure 2). Therefore, reprogramming features of survival mechanisms induced by antigenic stimulation are conserved in naïve and memory CD8^+^ T cells.

### Proliferation kinetics in response to αCD3 are unchanged in memory T cells

In naïve T cells, activation induces an autonomous program of cell division, return to quiescence (division destiny) and contraction *via* apoptosis. These kinetics are in turn modified by additional stimuli, cytokines and costimulation, but the same model can be used to measure the responses.^27^ The automated return to quiescence is a key feature regulating the size of the division burst^31^, ^32^, ^35^ that is appropriately modeled as a timed process.^27^, ^30^ We used quantitative methods to compare the responses of naïve and memory cells stimulated *in vitro* with plate‐bound αCD3. Mouse IL‐2 neutralizing monoclonal antibody S4B6 was added to the cultures to remove any potential downstream consequences of the more rapid production of IL‐2 by memory T cells.^38^

We first compared memory OT‐I CD8^+^ T cells isolated 4 months after *Listeria‐OVA* infection with naïve endogenous CD44^low^ CD8^+^ T cells isolated from the same mice, as they would have experienced the same inflammatory environment during the *Listeria‐OVA* infection.

**Figure 3:**
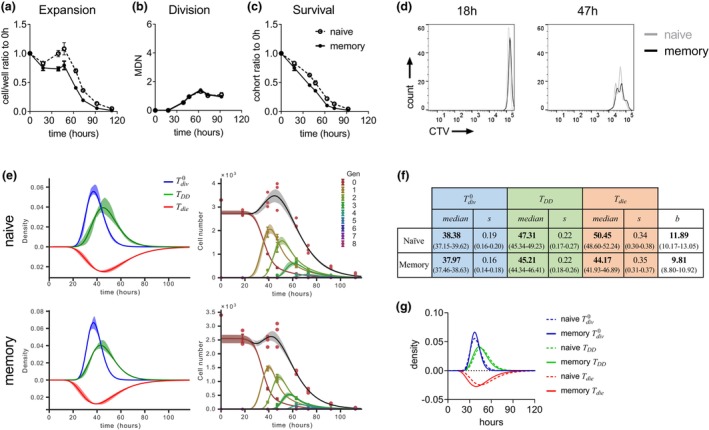
Proliferation kinetics of αCD3‐stimulated naïve and memory T cells. Memory OT‐I CD8^+^ T cells were isolated 4 months after transfer into C57BL/6 mice and infection with *Listeria‐OVA*. Naïve endogenous CD44^low^ CD8^+^ cells T cells were isolated from the same mice. Cells were labeled with the division tracking dye CellTrace Violet (CTV) and activated with plate‐bound αCD3. Endogenously produced interleukin (IL)‐2 was neutralized with mouse IL‐2–binding antibody S4B6. **(a)** Total cell number, **(b)** mean division number and **(c)** total cohort number of naïve and memory T cells over time. **(d)** CTV division plots of naïve (gray lines) and memory (black lines) T cells measured at 18 and 47 h after the start of culture. **(e)** Estimated Cyton2 distribution fitting overlaid with the model extrapolation and 95% confidence band from bootstrapping for naïve and memory T‐cell populations of $T_{\mathrm{div}}^{0}$ (blue lines), $T_{\mathrm{DD}}$ (green lines) and $T_{\mathrm{die}}$ (red lines) (left panels) and per generation (right panels). **(f)** Parameters from Cyton2 model fitting. Values shown are median (=*e* ^*m*^), standard deviation *s* and subsequent division time *b* with 95% confidence intervals in parentheses. **(g)** An overlay of distribution for time to first division $T_{\mathrm{div}}^{0}$, division destiny time $T_{\mathrm{DD}}$ and death times $T_{\mathrm{die}}$ of naïve and memory T cells using parameters from **e** and **f**. Data in **a–c** are presented as means ± standard error of the mean of triplicate cultures.

Stimulation with αCD3 alone induced a weak proliferative response in both naïve and memory T cells (Figure 3a). Applying precursor cohort methods^30^, ^31^, ^32^, ^33^ revealed that cells underwent an average of about two rounds of division before stopping division (Figure 3b). Hence, similar to the regulation of naïve T cells, memory T cells follow an automated program of return to quiescence. Furthermore, the kinetics of division (Figure 3b) and survival (Figure 3c) of both cell types were remarkably similar. Importantly, the rate at which the cells divided seemed indistinguishable (Figure 3b, d). We applied the Cyton2 mathematical model^27^ to CellTrace Violet (CTV) time series data to determine and compare the underlying kinetics of cell division and death (Figure 3e, f and Supplementary figure 3a). In this model, time to first division ($T_{\mathrm{div}}^{0}$), division rate (*b*), division destiny time ($T_{\mathrm{DD}}$) and time to die ($T_{\mathrm{die}}$) are independently regulated parameters. These fates are heterogeneous across a cell population, but times to division destiny and cell death are inherited within clonal families.^27^, ^30^ Overlaying distributions of time to first division, time to division destiny and survival times of memory and naïve T cells (Figure 3f) clearly confirmed that proliferation kinetics in the two cell types were very similar (Figure 3g) and that under these controlled *in vitro* conditions, memory T cells did not display a faster or enhanced proliferative response to αCD3 stimulation.

### Response to CD28 costimulation is reduced in memory T cells

**Figure 4:**
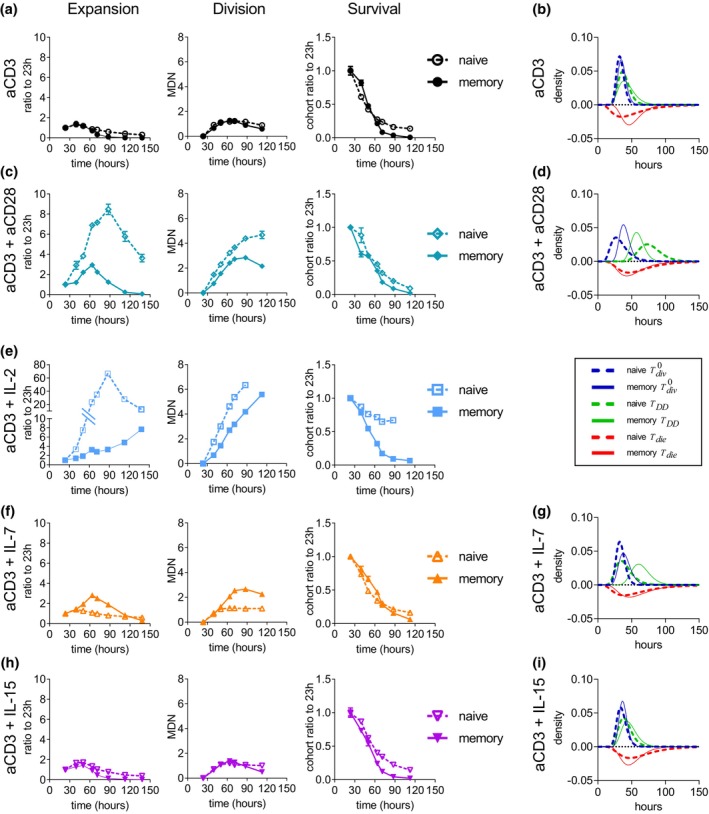
Sensitivity of αCD3‐stimulated naïve and memory CD8^+^ T cells to αCD28, interleukin (IL)‐2 and IL‐7 is altered in memory CD8^+^ T cells. Memory OT‐I CD8^+^ T cells were isolated 4 months after transfer into C57BL/6 mice and infection with *Listeria‐OVA*. Naïve OT‐I CD8^+^ T cells were isolated from nonimmunized mice. Cells were CellTrace Violet labeled and cultured with plate‐bound αCD3 in **(a, b)** complete media alone or in the presence of **(c, d)** 2 μg mL^−1^ αCD28; **(e)** 10 U mL^−1^ recombinant human IL‐2; **(f, g)** 1 ng mL^−1^ IL‐7 or **(h, i)** 1 ng mL^−1^ IL‐15. The αmIL‐2 monoclonal antibody clone S4B6 (25 μg mL^−1^) was added to all cultures. To compare the proliferative response of activated cells without confounding effects of differential cell loss early in the culture, cell numbers were normalized to surviving cells at 23.5 h for each condition. **(b, d, i)** Estimated Cyton2 distribution fitting of time to first division $T_{\mathrm{div}}^{0}$, division destiny time $T_{\mathrm{DD}}$ and death times $T_{\mathrm{die}}$. Best‐model fit data and 95% confidence intervals are shown in Supplementary figure 3b, and values are given in Supplementary table 1. Data in **a, c, e, f, h** are presented as means ± standard error of the mean of triplicate cultures. MDN, mean division number.

We next compared how additional signals are integrated. In naïve T cells, CD28 signaling provides a strong costimulatory signal and promotes T‐cell expansion both indirectly through enhancement of IL‐2 production^39^ and directly by induction of survival^39^ and increase of division destiny.^30^, ^31^ However, the requirement and importance of CD28 signaling in the expansion of memory T cells is less well established and have been a matter of debate.^24^, ^26^ The previous experiment compared endogenous naïve CD8^+^ T cells with OT‐I TCR transgenic CD8^+^ memory T cells. Here, we compared memory and naïve OT‐I T cells to avoid any potential differences that may arise between CD8^+^ T cells from OT‐I and C57BL/6 mice. Response to αCD3 was again equivalent (Figure 4a, b, Supplementary figure 3b, Supplementary table 1). The addition of αCD28 increased the expansion in both cell types (Figure 4c), verifying that memory T cells can receive and process costimulatory signals. Analysis of division kinetics revealed that the main effect of CD28 costimulation was on division destiny as has been previously observed with peptide‐stimulated naïve CD8^+^ OT‐I T cells.^30^, ^31^ However, the response magnitude was reduced in memory compared with naïve T cells (Figure 4c). Cyton2 modeling confirmed a difference in division destiny time by approximately 18 h between naïve and memory T cells (Figure 4d, Supplementary figure 3b, Supplementary table 1). As endogenously produced IL‐2 was neutralized and no IL‐2 was added to the culture, these differences were not a consequence of enhanced production or altered sensitivity to IL‐2. Hence, while memory CD8^+^ T cells respond to costimulation with αCD28, they are less sensitive than their naïve counterpart to that signal.

### Sensitivity to cytokine signals is altered in memory T cells

The enhanced response of resting memory T cells to IL‐2 and IL‐15 (Figure 1 and Supplementary figures 1, 4) raised the possibility that sensitivity to certain cytokines had been altered permanently and therefore could affect the response to antigenic stimulation. In this context, IL‐2 was of particular interest owing to its strong proliferation‐promoting potential and its rapid production after T‐cell activation.

The addition of IL‐2 increased expansion in both cell types; however, surprisingly, this response was greatly diminished in memory compared with naïve T cells (Figure 4e). As cells had divided beyond the resolution limit of the division tracking dye, insufficient division information for the later time points was available to apply Cyton2 modeling. However, precursor cohort analysis of early time points identified slower division and rapid cell death accounting for the reduced expansion of memory T cells (Figure 4e). Thus, the enhanced response to IL‐2 of resting memory T cells (Figure 1) appeared to be lost after T‐cell activation.

T‐cell receptor engagement renders both naïve^37^ and memory T cells (Figure 2) insensitive to IL‐7–induced survival signals and this was maintained throughout the duration of the culture with both cell types dying at a similar rate as in response to αCD3 stimulation alone (Figure 4a, f, Supplementary figure 3b, Supplementary table 1). Surprisingly, however, IL‐7 had a proliferation‐promoting effect on αCD3‐stimulated memory T cells that was not observed in naïve T cells (Figure 4f). Of note, neither the time it takes for the cells to start dividing nor the division rate was altered by IL‐7, instead the time that the cells divide before they reach division destiny was increased (Figure 4f, g, Supplementary figure 3b, Supplementary table 1). Thus, stimulation of memory T cells with αCD3 has divergent effects on IL‐7–mediated signals. Survival‐promoting features of IL‐7 are lost during the transition from resting to activated naïve and memory T cells (Figure 2); however, IL‐7 promotes cell division in activated memory but not in naïve T cells (Figure 4f, g).

In contrast, memory T cells remained largely unresponsive to IL‐15 after αCD3 stimulation, with no effect on survival or division observed (Figure 4h, i, Supplementary figure 3b, Supplementary table 1).

Although IL‐2 was neutralized in these experiments, disparities in activation‐induced production of other autocrine effector molecules in naïve and memory T cells could potentially have downstream effects on the responding T cells and therefore might explain response differences observed in these experiments. However, the same results were obtained in a repeat experiment where memory and naïve OT‐I CD8^+^ T cells were cocultured in the same culture wells to provide identical exposure to any effector molecules potentially produced by either cell type (Supplementary figure 5, Supplementary table 2).

Thus, despite the intrinsic proliferative response to TCR activation remaining comparable to naïve T cells, the response to costimulation and cytokines is altered in activated memory cells. Furthermore, a clear reprogramming of the response to IL‐2, IL‐7 and IL‐15 is observed between resting and antigen‐stimulated memory CD8^+^ T cells.

## DISCUSSION

When cultured, T‐cell responses can be measured and compared with accessible quantitative techniques to assess the underlying controls over division and survival. Applying these methods previously, we identified that naïve T‐cell activation follows a model of operation where internal timers governing division and death are acted upon directly by signal engagement. Further, we identified that cells can carry times to fate through generations, in part by the heritability of changes in the production rate of controlling proteins, such as Myc.^27^, ^30^ We reasoned that these methods provided a foundation for comparing the quantitative performance of naïve and memory cells exposed to similar signals under controlled conditions *in vitro*. This question was of interest as there has been an ongoing debate as to the activation and costimulation dependence that might be different between the two populations. Here we report that, despite the many changes that T cells undergo during the transition from the resting naïve state through cell expansion and back to memory, the fundamental activation‐induced fate programs remain similar for naïve and memory cells, albeit with notable quantitative differences. Specifically, the reprogramming of survival coupled to an autonomous division burst followed by a return to quiescence is preserved in memory T cells. The division times of naïve and memory cells during the proliferative response after polyclonal stimulation with aCD3 antibodies are also similar. However, memory T cells have acquired different sensitivities to signals from cytokines and costimulation, both when resting and after activation. As expected, sensitivities to homeostatic cytokines were enhanced for resting memory T cells. By contrast and perhaps counterintuitively, when stimulated, the proliferative response to cytokine and costimulatory signals in antigen‐stimulated memory T cells was shifted in a negative direction in the memory cells compared with naïve cells.

Maintaining a long‐lived pool of resting naïve and antigen‐experienced T cells is essential for the ability to respond to immune challenges. It is well established that memory T cells are less restricted than naïve T cells in their reliance on homeostatic survival signals, responding to additional factors such as IL‐15.^34^ Here we found that IL‐2, a factor best known for its effect on activated T cells, not only induced survival but also promoted cell division in resting memory but not naïve T cells, suggesting an additional role for IL‐2 in the maintenance of memory T‐cell populations. The expansion of resting memory CD8^+^ T cells in response to IL‐2 observed here is consistent with reported bystander activation of memory CD8^+^ T cells in viral infections or IL‐2 cytokine therapy.^40^, ^41^, ^42^ As CD25 is not expressed by resting memory T cells and is not upregulated in bystander‐activated CD8^+^ memory T cells^40^ enhanced signaling by the low‐affinity receptor for IL‐2 may be mediating this effect. Studies that reported similar results for naïve T cells *in vitro* ^43^ and *in vivo* ^44^, ^45^ used much higher levels of IL‐2, pointing to a quantitative shift in the processing of this signal by the cells.

Identical response kinetics in naïve and memory T cells induced by TCR signaling observed here are consistent with previous reports showing similar proliferation rates and equal expansion capacity^15^, ^38^, ^46^ and therefore are pointing to other mechanisms for the greater and faster recall response achieved. Apart from the quantitative advantage of increased precursor frequency of cells specific for a particular antigen, several other mechanisms are likely to contribute. Although our results demonstrate equal response kinetics to the relatively strong stimulus provided by plate‐bound αCD3, this did not address potential differences in thresholds for TCR signal strength required for activation. A reduction in these thresholds, as has been proposed^21^, ^47^ could contribute to memory T cells getting activated more readily.

Increased and more rapid production of effector molecules such as IL‐2 by memory T cells may potentially explain observations of a greater expansion of memory T cells *in vitro*.^10^, ^22^ Further, the inflammatory environment resulting from an immune response *in vivo* may contribute to differences in response rates.^48^ A higher sensitivity or increased response to other signals not tested here may mediate differences in *in vivo* response rates. By contrast, reduced responses to key activating signals, IL‐2 and aCD28, may explain the preferential expansion of naïve T cells in other experimental systems,^15^, ^49^, ^50^ highlighting the importance of isolating the contribution of these signals. The ability to carefully measure and quantify immune cell response dynamics under controlled conditions is of particular value for studies aiming to measure the responsiveness of human naïve and memory cells and compare sensitivity and performance between individuals and those susceptible to immune dysfunction. As the environment that T cells are exposed to during an infection is a complex mixture of signals constantly changing over time and space, assessing the response of naïve and memory T cells to the canonical signals that these cells may encounter and understanding the mode and consequence of the integration offer important insights into the logical operation of the system and how it might be manipulated.

## METHODS

### Mice

C57BL/6, C57BL/6‐Ly5.1 and OT‐I TCR transgenic mice expressing TCRs specific for SIINFEKL sequence from chicken ovalbumin were obtained from the Walter and Eliza Hall Institute (Parkville, VIC, Australia) animal facility (Kew, VIC, Australia). Mice were maintained under specific pathogen‐free conditions and used between 8 weeks and 6 months of age. All animal experiments were performed under the approval of the Walter and Eliza Hall Institute Animal Ethics Committee.

### *In vivo* induction of T‐cell memory

CD8^+^ T cells were isolated from nonimmunized OT‐I‐Ly5.1 or OT‐I mice using the EasySep Mouse CD8^+^ T cell Isolation Kit (STEMCELL Technologies, Vancouver, British Columbia, Canada) and transferred to C57BL/6 or C57/BL6‐Ly5.1 host mice, respectively. One day later, host mice received 2500 colony‐forming units of recombinant *Listeria monocytogenes*–expressing OVA_257–264_ (*Listeria‐ova*) intravenously. *Listeria‐OVA* expresses a secreted form of OVA.^51^ Frozen stocks of recombinant *Listeria‐OVA* were grown in brain–heart infusion broth supplemented with 5 mg mL^−1^ erythromycin (Sigma‐Aldrich, Sydney, NSW, Australia). At the mid‐log growth phase, culture samples were measured by optical density and diluted in phosphate‐buffered saline for injection into mice *via* the lateral tail vein. Bacterial counts were verified by plating culture dilutions on brain–heart infusion agar plates and incubating overnight.

### T‐cell isolation

OT‐I memory T cells were isolated 10–22 weeks after infection with *Listeria‐OVA* from lymph nodes (inguinal, axillary, brachial and superficial cervical) and spleens using negative isolation by EasySep Mouse CD8^+^ T Cell Isolation Kit (STEMCELL Technologies) followed by labeling with anti‐CD45.1 (Ly5.1)‐APC antibodies (BD Biosciences, San Jose, CA, USA) and positive enrichment using the EasySep APC selection kit (STEMCELL Technologies). Purity was typical > 95% CD8^+^ CD45.1^+^.

In experiments where naïve CD8^+^ T cells from the same host mice were used, first OT‐I memory T cells were isolated as described above. Naïve CD44^low^ T cells were then purified from the CD8^+^CD45.1^neg^ fraction by labeling with anti‐CD44‐APC antibodies (BD Biosciences), followed by separation into CD44^high^ and CD44^low^ populations using the EasySep APC selection kit (STEMCELL Technologies). Purity was > 95% CD8^+^CD44^low^CD45.1^neg^.

For the reverse transcription quantitative PCR (RT‐qPCR) analysis, endogenous CD44^+^CD8^+^ memory T cells were isolated from C57BL/6 mice 11 weeks after infection with *Listeria‐OVA* using negative isolation by EasySep Mouse CD8^+^ T Cell Isolation Kit (STEMCELL Technologies) followed by labeling with anti‐CD44‐APC and positive enrichment using the EasySep APC selection kit (STEMCELL Technologies). Purity was 80% CD44^+^CD8^+^.

Naïve OT‐I CD8^+^ T cells were isolated from nonimmunized OT‐I or OT‐I‐Ly5.1 mice using negative isolation by EasySep Mouse CD8^+^ T Cell Isolation Kit (STEMCELL Technologies). Purity was typically > 95% CD8^+^Vα2^+^.

### CTV labeling

For analysis of cell division, T cells were labeled with 5 μM CTV (Invitrogen, Carlsbad, CA, USA) for 20 min at 37°C in sterile phosphate‐buffered saline containing 0.1% bovine serum albumin (Sigma, St Louis, MI, USA).

In a coculture experiment of naïve and memory T cells, naïve T cells were labeled with 5 μM carboxyfluorescein succinimidyl ester (Invitrogen) for 10 min at 37°C in sterile phosphate‐buffered saline containing 0.1% bovine serum albumin to distinguish from CTV labeled memory T cells.

### *In vitro* T‐cell culture

T cells were cultured in complete medium made of RPMI (Roswell Park Memorial Institute)‐1640, supplemented with 10% (vol/vol) FBS, 10 mM HEPES [4‐(2‐hydroxyethyl)‐1‐piperazineethanesulfonic acid], 100 U mL^−1^ penicillin, 100 μg mL^−1^ streptomycin, 2 mM GlutaMAX, 1 mM non‐essential amino acids, 1 mM sodium pyruvate (all Invitrogen) and 50 μM β‐2‐mercaptoethanol (Sigma).

For proliferation and survival assays, T cells were plated in flat‐bottomed 96‐well tissue culture plates at 3 × 10^3^ to 1 × 10^4^ cells/well. In the coculture proliferation experiment, 3.3 × 10^3^ cells of each cell type were added per well.

For CD3 antibody stimulation, tissue‐culture wells were coated with 10 μg mL^−1^ anti‐CD3 antibody (clone 145‐2C11; Walter and Eliza Hall Institute monoclonal antibody facility) before culture. In the coculture experiment, 4 μg mL^−1^ anti‐CD3 antibody was used. The mouse IL‐2–neutralizing antibody clone S4B6 (Walter and Eliza Hall Institute monoclonal antibody facility) was added to all cultures at 25 μg mL^−1^.

Recombinant mouse IL‐7 (PeproTech, Rocky Hill, NJ, USA), recombinant human IL‐2 (PeproTech) or recombinant mouse IL‐15 (Shenandoah Biotechnology, Elverson, PA, USA) were added at the concentrations indicated.

### Cell counting

At time points indicated, 10^4^ beads (Rainbow calibration particles; BD Biosciences) were added to tissue culture wells immediately before analysis, and the ratio of beads to live cells was used to calculate the absolute cell number in each sample. Propidium iodide (0.2 μg mL^−1^, Sigma) was used for dead‐cell exclusion.

To distinguish between naïve (CD45.2) and memory (CD45.1) OT‐I T cells in the coculture experiment, an anti‐CD45.1‐APC antibody (BD Biosciences) was added to the bead suspension.

Flow cytometry was performed on FACSCanto II or Fortessa X‐20 cytometer (both BD Biosciences). Data were analyzed using FlowJo software (Tree Star, Ashland, OR, USA).

### Calculation of cell expansion, survival and mean division number (MDN)

The total cohort number, which estimates the survival of the starting population, and the MDN were calculated using a modification of the precursor cohort methods described previously.^30^, ^31^, ^32^, ^33^ This method removes the effect of cell division on the total cell numbers by dividing the cell number per division by two to the power of the division number to obtain a cohort number for that division (*C*_*i*_).

$$C_{i}=\frac{\text{number of cells in division},i}{2^{i}}$$

The “total cohort number” at any given time point is the sum of all *C*_*i*_ at this time point, with *n* being the highest division measured.

$$\text{total cohort number}=\sum_{i=0}^{n}C_{i}$$

If no cell death occurred during the culture, the total cohort number would remain equal to the starting cell number. The shape of the total cohort over time provides information about the rate of cell loss over time.^30^

In the modified cohort method,^31^ MDN, which represents the average number of divisions the starting cell population has undergone at a given time point, is calculated as follows:

$$\mathrm{MDN}=\frac{\sum_{i=0}^{n}i\cdot C_{i}}{\sum_{i=0}^{n}C_{i}}$$

Plots of MDN over time can be used to estimate proliferation features, including the time to first division, division rates and division destiny.^30^, ^31^, ^32^, ^33^

A maximum number of seven to eight divisions can be traced using cell division tracking dyes such as carboxyfluorescein succinimidyl ester and CTV. When cells cannot be allocated to division peaks (i.e. > 8 divisions), total cohort number and MDN cannot be calculated accurately. Data points where this has occurred are not included in the analysis.

To compare expansion and survival curves between different cell populations, the total cell number or total cohort number, respectively, was divided by the starting cell population. In some cases, expansion and survival were calculated as the ratio to the number of live cells measured at approximately 24 h after activation, to remove the effect of differences in cell death after stimulation between different conditions and cell types.

The proportion of cells of the starting populations that have undergone at least one division was calculated as follows:

$$\text{Proportion of starting population divided}=\frac{\text{total cohort number}-C_{i=0}\;}{\text{starting population}}$$

### Unstimulated cell survival

Lymphocyte survival of unstimulated cells varies and conforms well to a log‐normal probability distribution function.^35^ We fitted a log‐normal survival curve, a complementary cumulative distribution function, to unstimulated data by minimizing the sum of squares. Typically, we present log‐normal probability distribution functions with parameters median (= *e* ^*m*^) and standard deviation *s*.

### Cyton2

For time series experiments a reduced version of the Cyton2 model^27^ was used to estimate and compare proliferation and survival features with seven parameters. To fit the reduced Cyton2 model to cell numbers per generation data acquired from flow cytometry, we adopted the strategy previously described.^27^ We let $\left\{n_{i,r}\left(t\right)\right\}$ be a collection of the measured cell numbers in division $i\in G=\left\{0,1,\ldots ,G\right\}$ at time point $t\in T=\left\{t_{j}\in {\mathbb{R}}_{\geq 0}:j=0,1,\ldots ,J\right\}$ with a replicate index $r\in R=\left\{0,1,\ldots ,R\right\}$, where $G,J\;\text{and}\;R\in {\mathbb{Z}}_{0+}$ are the maximum number of generation that can be traced, the last harvested time point and total number of replicates, respectively, in a given experiment. In particular, we estimated log‐normally distributed random variables that represent time to first division $\left(T_{\mathrm{div}}^{0}\sim \mathrm{LN}\left(m_{\mathrm{div}}^{0},s_{\mathrm{div}}^{0}\right)\right)$, time to division destiny $\left(T_{dd}∼LN(m_{dd},s_{dd})\right)$ and time to death $\left(T_{\mathrm{die}}\sim \mathrm{LN}\left(m_{\mathrm{die}},s_{\mathrm{die}}\right)\right)$ for recapitulating kinetics of the stimulated cells. In addition, we estimated a constant $b\in {\mathbb{R}}_{\geq 0}$ that captures an average time for cells to traverse subsequent generations after the first division. This use of a deterministic time for *b* accounts for the reduction of one parameter from the full probabilistic version of Cyton2 and improves the robustness of fitting for lymphocyte proliferation experiments.^27^ In summary, we have a total of seven parameters to estimate, $\theta =\left(m_{\mathrm{div}}^{0},s_{\mathrm{div}}^{0},m_{\mathrm{dd}},s_{\mathrm{dd}},m_{\mathrm{die}},s_{\mathrm{die}},b\right)$, for a given data set. We define the following residual sum‐of‐squares (RSS) as our cost function:

(1) $$L\left(\theta \right)=\sum_{t\in T\backslash \left\{t_{0}\right\}}\sum_{i\in G}\sum_{r\in R}{\left(n_{i,r}\left(t\right)-f_{i}\left(t;\theta \right)\right)}^{2},$$

where $f_{i}\left(t\right)$ is the model prediction to utilize the least‐squares method and the Levenberg–Marquardt optimization algorithm^52^ to find an approximate minimum,

$$\left\{\theta^{*}\right\}\in \underset{\theta }{\arg\;\min}\;L\left(\theta \right),$$

$$s.t.m_{\mathrm{div}}^{0},m_{\mathrm{dd}},m_{\mathrm{die}}\in \left(0,500\right];s_{\mathrm{div}}^{0},s_{\mathrm{dd}},s_{\mathrm{die}}\in \left(0,2\right];b\in \left[0,100\right].$$

In Equation 1, we excluded cell numbers at the first time point (i.e. $t_{0}=0h$) in the fitting procedure to minimize the effect of unstimulated cell death. By doing so, we aimed to maximize the accuracy of the parameter estimates specifically for dividing cells.

To explore the parameter space, we prescribed 100 sets of initial parameter guesses as required by the algorithm, and their values were drawn uniformly at random from the defined parameter ranges. We recorded the RSS for each set and identified the best‐fitted parameters based on the corresponding lowest RSS values. After obtaining the best fit, we performed the bootstrap method^53^ to compute uncertainties around the point estimates. To do so, we constructed an artificial data set by resampling with replacements for each time point from the original data. This process was repeated 100 times, resulting in an additional 100 estimates. These estimates allowed us to calculate 95% confidence intervals for each parameter, as well as confidence bands for the model predictions of cell numbers.

### Reverse transcription quantitative PCR

For analysis of gene expression by qRT‐PCR, memory T cells were cultured at 1 × 10^6^ cells/well in a 48‐well plate, uncoated or coated with 20 μg mL^−1^ anti‐CD3 antibody (clone 145‐2C11) before culture. For survival assay setup in parallel, cells were stimulated and cultured in 96‐well plates at 1 × 10^4^ cells/well.

Recombinant mouse IL‐7 (PeproTech) or recombinant mouse IL‐15 (Shenandoah Biotechnology) was added at concentrations indicated.

The total RNA was isolated using the RNAeasy Kit including the DNase digestion step (Qiagen, Venlo, The Netherlands) and transcribed to cDNA using High Capacity RNA to cDNA Mastermix (Applied Biosystems, Waltham, MA, USA), following the manufacturer's instructions. Quantitative PCR was performed using Power SYBR green Mastermix on an ABI‐PRISM 7900 thermal cycler (all Applied Biosystems). The relative messenger RNA level of samples is expressed relative to Hydroxymethylbilane synthase (HMBS). The primers used were as follows:

*a1* (5′‐GTCATACTTGGATGACTTTCACGTG, ATTCTCCTGTGTTATTCATTATGAATTCTG‐3′);

*bcl2* (5′‐TTATAAGCTGTCACAGAGGGGCTAC, GAACTCAAAGAAGGCCACAA‐3′);

*bcl‐xL* (5′‐TGGAGTCAGTTTAGTGATGTCGAAG, AGTTTACTCCATCCCGAAAGAGTTC‐3′);

*HMBS* (5′‐CCTGGTTGTTCACTCCCTGA, CAACAGCATCACAAGGGTTTT‐3′).

## AUTHOR CONTRIBUTIONS

**Susanne Heinzel:** Conceptualization; data curation; formal analysis; funding acquisition; investigation; methodology; visualization; writing – original draft; writing – review and editing. **HoChan Cheon:** Formal analysis; methodology. **Gabrielle T Belz:** Data curation; resources. **Philip D Hodgkin:** Conceptualization; formal analysis; funding acquisition; investigation; methodology; resources; writing – review and editing.

## CONFLICT OF INTEREST

The authors declare no conflict of interest.

## Supporting information


Supplementary figure 1
Supplementary figure 2
Supplementary figure 3
Supplementary figure 4
Supplementary figure 5
Supplementary table 1
Supplementary table 2

## Data Availability

The data that support the findings of this study are available from the corresponding author upon reasonable request.
