# Supplementary material for: Survival and division fate programs are preserved but retuned during the naïve to memory CD8+ T‐cell transition
Source: Immunol Cell Biol. 2023 Oct 15;102(1):46–57. doi: 10.1111/imcb.12699 (PMC10952575; doi:10.1111/imcb.12699)
Supplement: Supplementary file 1 — Supplementary figure 1 Supplementary figure 2 Supplementary figure 3 Supplementary figure 4 Supplementary figure 5 Supplementary table 1 Supplementary table 2 [file IMCB-102-46-s001.pdf]

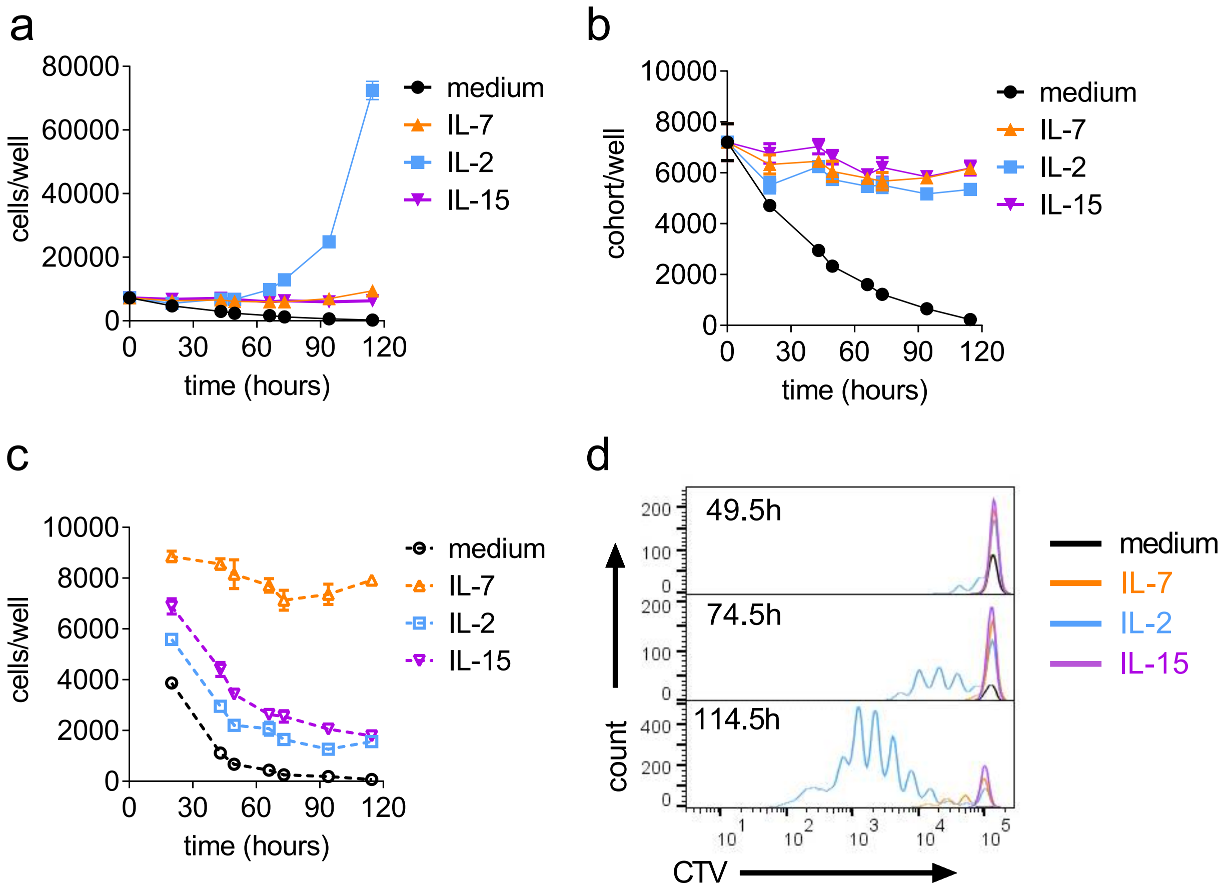

SUPPLEMENTARY FIGURE 1: Response to IL-15 by memory CD8$^+$ T cells is similar to IL-7.

Memory OT-I CD8$^+$ T cells (filled symbols) were isolated 11 weeks post transfer into C57BL/6 mice and infection with Listeria-ova, CTV labelled and cocultured with CFSE labelled naive CD8$^+$ T cells isolated from non-immunised C57BL/6 mice. Cells were cultured in complete media alone, or in the presence of 100U/mL rhIL-2, 5ng/mL IL-7 or 5ng/mL IL-15. **(a)** Total cell number and **(b)** Total cohort number of memory CD8$^+$ T cells over time. **(c)** Total naive CD8$^+$ T cells over time. **(d)** CTV division plots of memory CD8$^+$ T cells measured at 49.5 hours, 74.5 hours and 114.5 hours after start of culture. Data in **(a-c)** are presented as means±s.e.m. of triplicate cultures.

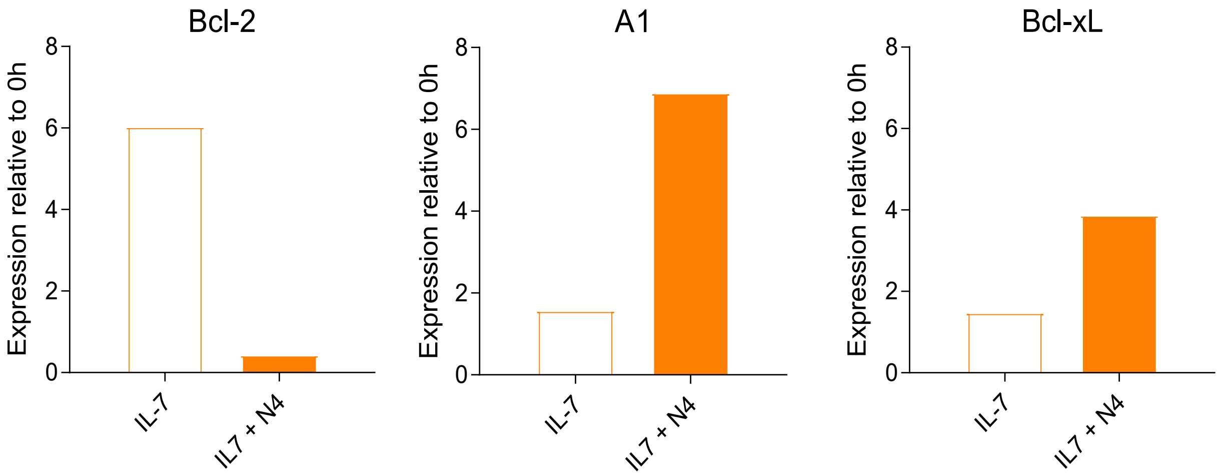

SUPPLEMENTARY FIGURE 2: TCR signalling induced repression of Bcl-2 production antigen stimulated OT-I memory CD8⁺ T cells.

qRT-PCR analysis of Bcl-2, Mcl-1, A1 and Bcl-xL mRNA of memory OT-I $CD8^+$ T cells cultured for 5 hours in complete media with 10ng/mL IL-7 alone or in the presence of 100ng/mL SIINFEKL peptide (N4). OT-I $CD8^+$ T cells were isolated 3 months post transfer into C57BL/6 mice and infection with Listeria-ova. Data are representative of 4 independent experiments.

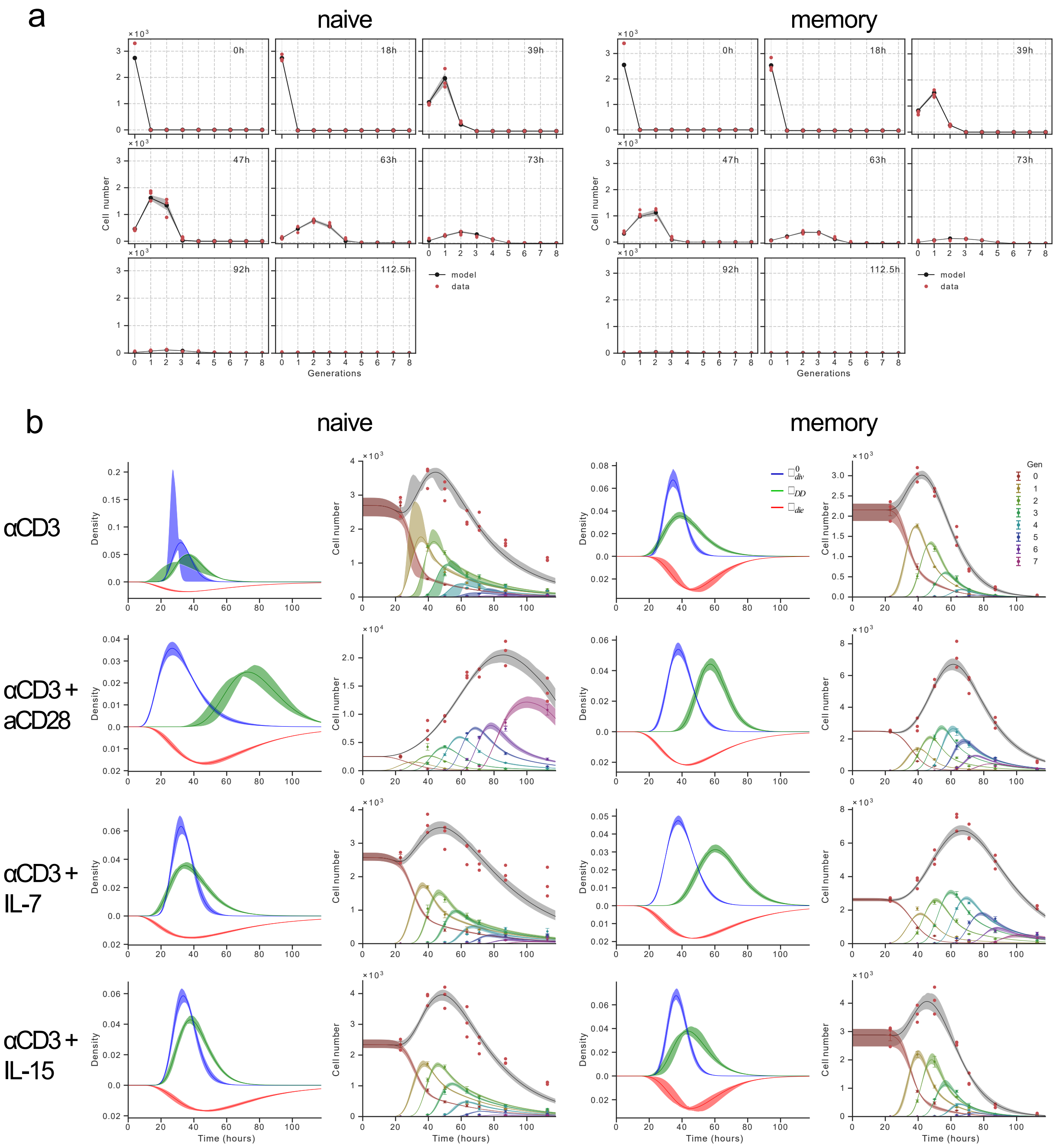

SUPPLEMENTARY FIGURE 3: Model fitting to stimulated naive and memory T cells. **(a)** Live cells per generation and the model extrapolation at harvested time points of data presented in Fig 3. **(b)** Model fits of data presented in Fig 4. Estimated Cyton2 distribution fitting overlaid with the model extrapolation and 95% confidence band from bootstrapping for naive and memory T cell populations of of T_{div}^{0} (blue lines), T_{DD} (green lines) and T_{die} (red lines) (left panels) and per division (right panels).

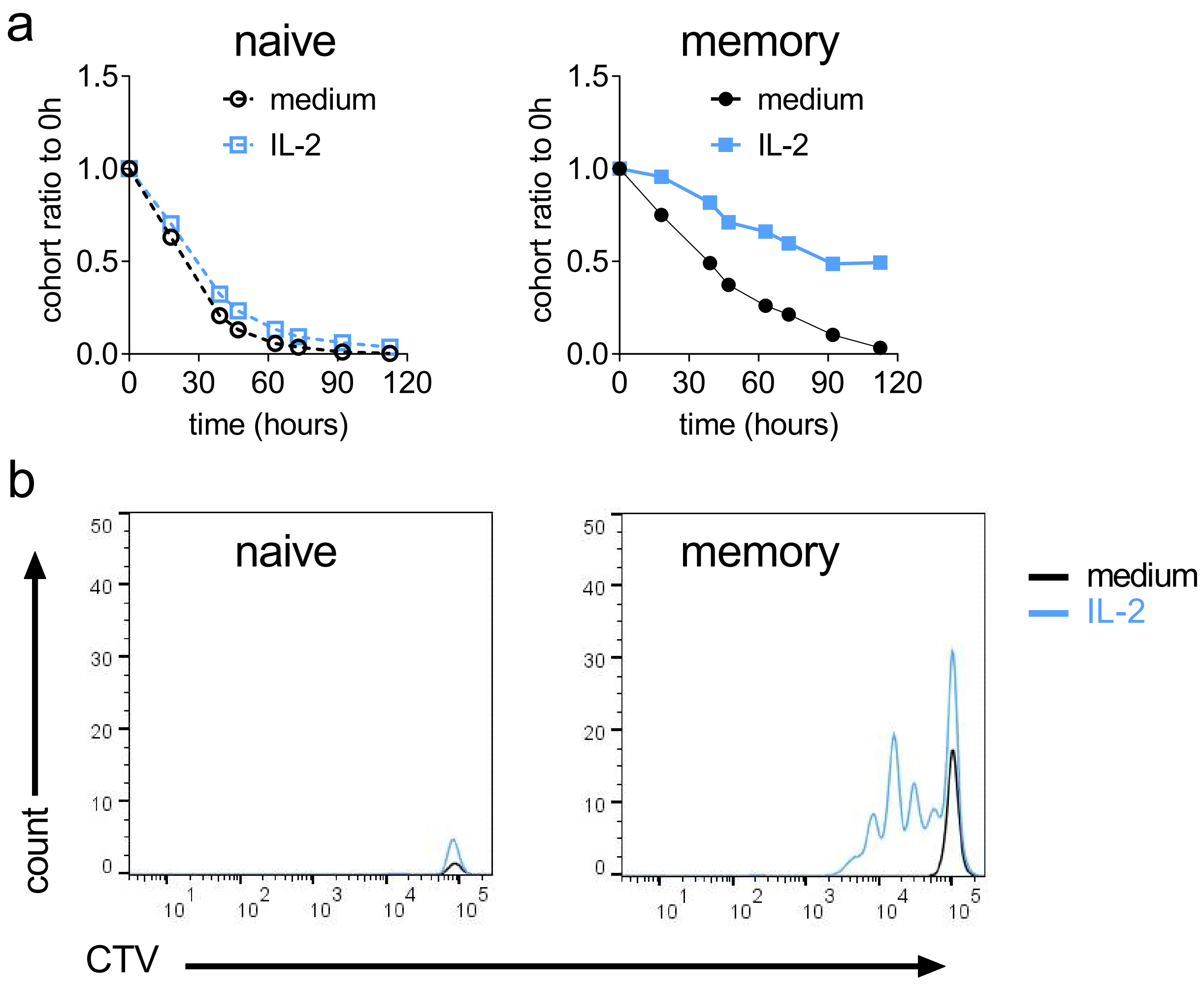

SUPPLEMENTARY FIGURE 4: 10U/mL rhIL-2 promotes survival and division in resting memory CD8$^+$ T cells.

Memory OT-I CD8$^+$ T cells (filled symbols) were isolated 4 months post transfer into C57BL/6 mice and infection with Listeria-ova. Naive endogenous CD44^{neg}CD8$^+$ cells T cells (open symbols) were isolated from the same mice. Cells were cultured in complete media alone (black lines), or in the presence of 10U/mL rhIL-2 (blue lines). **(a)** Proportion of cohort number compared to starting population over time of cells cultured in medium alone or with IL-2. **(b)** Representative CTV division plots measured at 73 hours after start of culture. Data in **(a)** are presented as means±s.e.m. of triplicate cultures.

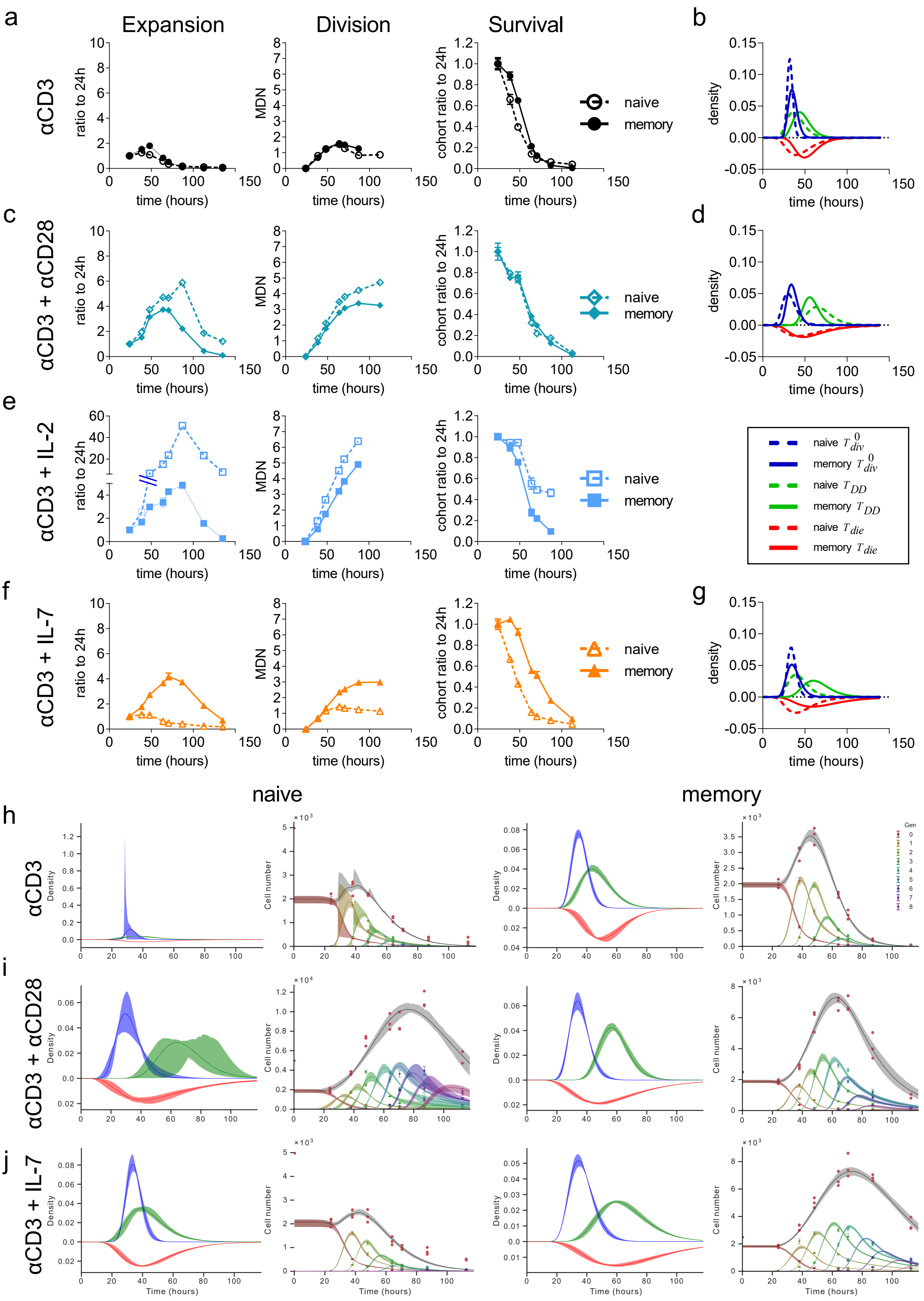

a
Expansion
Division
Survival
αCD3
ratio to 24h
MDN
cohort ratio to 24h
time (hours)
naive
memory
b
density
c
αCD3 + αCD28
d
e
αCD3 + IL-2
naive T_{div}^{0}
memory T_{div}^{0}
naive T_{DD}
memory T_{DD}
naive T_{die}
memory T_{die}
f
αCD3 + IL-7
g
naive
memory
h
i
j
Density
Cell number
Time (hours)
Gen

SUPPLEMENTARY FIGURE 5: Coculture of naive and memory CD8$^+$ T cells does not alter proliferation kinetics

Memory OT-I CD8$^+$ T cells (filled symbols) were isolated 5 months post transfer into C57BL/6 mice and infection with Listeria-ova, CTV labelled and cocultured with CFSE labelled naive OT-I CD8$^+$ T cells isolated from non-immunised mice (open symbols). Cells were cultured with plate bound αCD3 in **(a,b,h)** complete media alone or in the presence of **(c,d,i)** 2μg/mL αCD28; **(e)** 10U/mL rhIL-2, or **(f,g,j)** 1ng/mL IL-7. 25μg/mL αmIL-2 mAb clone S4B6 was added to all cultures. Cell numbers were normalised to surviving cells at 24 hours for each condition. **(b,d and g)** estimated Cyton2 distribution fitting of T_{div}^{0} T_{DD} and T_{die} **(h-j)** Estimated Cyton2 distribution fitting overlaid with the model extrapolation and 95% confidence band from bootstrapping for naive and memory T cell populations of T_{div}^{0} (blue lines), T_{DD} (green lines) and T_{die} (red lines) (left panels) and per division (right panels) of data from **(a,c,f)**. Best model fit data and 95% confidence intervals are given in Supp. Table II. Data in **(a,c,e,f)** are presented as means±s.e.m. of triplicate cultures.

SUPPLEMENTARY TABLE I:

Cyton 2 modelling best fit results from data presented in Fig 4. Values shown are median (=e^{m}), standard deviation *s* and subsequent division time *b* with 95% confidence intervals in brackets

		T_{div}^{0}		T_{DD}		T_{die}		
		median	*s*	*median*	*s*	*median*	*s*	*b*
aCD3	Naïve	**33.01** (27.25-34.20)	0.17 (0.07-0.19)	**37.95** (32.18-39.32)	0.22 (0.20-0.39)	**47.38** (45.38-51.23)	0.55 (0.52-0.57)	**8.60** (8.36-11.53)
	Memory	**35.49** (34.64-36.21)	0.17 (0.15-0.19)	**41.81** (40.09-43.53)	0.28 (0.25-0.30)	**49.84** (47.79-52.95)	0.28 (0.24-0.31)	**10.59** (9.83-10.93)
aCD3 + aCD28	Naïve	**31.10** (30.22-32.33)	0.39 (0.36-0.42)	**76.36** (71.68-80.85)	0.21 (0.18-0.25)	**57.15** (56.00-58.83)	0.47 (0.44-0.50)	**10.02** (9.86-10.19)
	Memory	**39.15** (38.52-39.66)	0.19 (0.18-0.21)	**58.58** (56.91-60.25)	0.16 (0.14-0.17)	**50.17** (49.05-51.07)	0.40 (0.38-0.42)	**7.76** (7.48-8.11)
aCD3 + IL-7	Naïve	**33.43** (32.55-34.70)	0.19 (0.17-0.21)	**38.03** (36.52-39.80)	0.31 (0.28-0.34)	**53.27** (51.17-55.66)	0.57 (0.54-0.61)	**10.62** (10.21-11.06)
	Memory	**39.44** (38.99-39.83)	0.22 (0.20-0.23)	**62.98** (61.46-64.96)	0.21 (0.19-0.22)	**56.18** (54.20-57.97)	0.43 (0.41-0.45)	**10.08** (9.88-10.31)
aCD3 + IL-15	Naïve	**34.87** (33.86-35.81)	0.20 (0.19-0.21)	**40.14** (38.97-41.40)	0.24 (0.22-0.26)	**58.35** (55.39-60.53)	0.45 (0.42-0.49)	**9.54** (8.95-9.91)
	Memory	**37.36** (36.48-38.18)	0.16 (0.15-0.17)	**45.94** (43.75-48.54)	0.24 (0.20-0.26)	**49.74** (47.06-53.80)	0.31 (0.25-0.34)	**9.59** (9.12-10.13)

SUPPLEMENTARY TABLE II:

Cyton 2 modelling best fit results from data presented in Supplementary Fig. S5. Values shown are median (=e^m), standard deviation *s* and subsequent division time *b* with 95% confidence intervals in brackets.

		T^0_{div}		T_{DD}		T_{die}		
		median	*s*	*median*	*s*	*median*	*s*	*b*
aCD3	Naïve	**32.23** (28.34-33.76)	0.10 (0.01-0.13)	**38.72** (34.35-41.80)	0.26 (0.24-0.31)	**44.32** (42.26-46.48)	0.35 (0.33-0.37)	**9.30** (8.90-9.93)
	Memory	**35.36** (34.50-36.14)	0.15 (0.14-0.16)	**45.93** (44.62-47.30)	0.22 (0.20-0.24)	**52.52** (50.30-54.83)	0.25 (0.22-0.28)	**10.30** (9.73-10.80)
aCD3 + aCD28	Naïve	**31.53** (31.02-32.62)	0.26 (0.19-0.36)	**66.99** (59.21-85.75)	0.21 (0.14-0.24)	**52.69** (49.43-56.98)	0.50 (0.40-0.59)	**9.51** (8.75-10.52)
	Memory	**34.85** (34.52-35.37)	0.18 (0.16-0.21)	**57.97** (56.42-60.35)	0.16 (0.15-0.17)	**56.35** (54.14-58.46)	0.41 (0.38-0.45)	**8.67** (8.30-9.18)
aCD3+ IL-7	Naïve	**34.31** (33.43-35.37)	0.15 (0.13-0.16)	**42.15** (40.25-44.68)	0.28 (0.25-0.32)	**45.14** (43.51-48.04)	0.38 (0.35-0.40)	**10.29** (9.92-10.78)
	Memory	**36.12** (35.32-37.20)	0.22 (0.20-0.23)	**63.70** (61.84-66.11)	0.25 (0.24-0.27)	**69.17** (66.31-71.59)	0.41 (0.37-0.46)	**11.33** (11.19-11.46)
